# Homeobox gene expression profile indicates *HOXA5* as a candidate prognostic marker in oral squamous cell carcinoma

**DOI:** 10.3892/ijo.2011.1321

**Published:** 2011-12-29

**Authors:** CAMILA OLIVEIRA RODINI, FLÁVIA CALÓ AQUINO XAVIER, KATIÚCIA BATISTA SILVA PAIVA, MARIA FERNANDA DE SOUZA SETÚBAL DESTRO, RAQUEL AJUB MOYSES, PEDRO MICHALUARTE, MARCOS BRASILINO CARVALHO, ERICA ERINA FUKUYAMA, HEAD and NECK GENOME PROJECT GENCAPO, ELOIZA HELENA TAJARA, OSWALDO KEITH OKAMOTO, FABIO DAUMAS NUNES

**Affiliations:** 1 Laboratory of Molecular Pathology, Department of Oral Pathology, School of Dentistry, University of São Paulo, Av. Prof. Lineu Prestes 2227, Cidade Universitária, 05508-000 São Paulo; 2 Department of Head and Neck Surgery, School of Medicine, University of São Paulo, Av. Dr. Arnaldo 455, Cerqueira César, 01246903 São Paulo; 3 Head and Neck Surgery Division, Heliópolis Hospital Complex, Rua Cônego Xavier 276, Sacomã, 04231-030 São Paulo; 4 Department of Head and Neck Surgery, Arnaldo Vieira de Carvalho Cancer Institute, Rua Dr Cesário Motta Junior 112, Vila Buarque, 01221020 São Paulo; 5 Author list and addresses presented in the Appendix; 6 Department of Molecular Biology, São José do Rio Preto School of Medicine, Av. Brigadeiro Faria Lima 5416-Vila São Pedro, 15090-000 São José do Rio Preto; 7 Department of Genetics and Evolutionary Biology, Institute of Biosciences, University of São Paulo, R. do Matão, travessa 14 321, Cidade Universitária, 05508-090 São Paulo; 8 Human Genome Research Center, Department of Genetics, Biosciences Institute, University of São Paulo, Rua do Matão, trav. 14 321, Cidade Universitária, 05508-090 São Paulo, Brazil

**Keywords:** oral cancer, homeobox genes, HOXA5, prognosis

## Abstract

The search for molecular markers to improve diagnosis, individualize treatment and predict behavior of tumors has been the focus of several studies. This study aimed to analyze homeobox gene expression profile in oral squamous cell carcinoma (OSCC) as well as to investigate whether some of these genes are relevant molecular markers of prognosis and/or tumor aggressiveness. Homeobox gene expression levels were assessed by microarrays and qRT-PCR in OSCC tissues and adjacent non-cancerous matched tissues (margin), as well as in OSCC cell lines. Analysis of microarray data revealed the expression of 147 homeobox genes, including one set of six at least 2-fold up-regulated, and another set of 34 at least 2-fold down-regulated homeobox genes in OSCC. After qRT-PCR assays, the three most up-regulated homeobox genes (*HOXA5*, *HOXD10* and *HOXD11*) revealed higher and statistically significant expression levels in OSCC samples when compared to margins. Patients presenting lower expression of *HOXA5* had poorer prognosis compared to those with higher expression (P=0.03). Additionally, the status of *HOXA5*, *HOXD10* and *HOXD11* expression levels in OSCC cell lines also showed a significant up-regulation when compared to normal oral keratinocytes. Results confirm the presence of three significantly upregulated (>4-fold) homeobox genes (*HOXA5*, *HOXD10* and *HOXD11*) in OSCC that may play a significant role in the pathogenesis of these tumors. Moreover, since lower levels of *HOXA5* predict poor prognosis, this gene may be a novel candidate for development of therapeutic strategies in OSCC.

## Introduction

Oral squamous cell carcinoma (OSCC) is the sixth most common cancer worldwide and often invades tissues locally and metastasizes to cervical lymph nodes (1–3). Oncogene overexpression or inactivation mechanisms on tumor suppressor genes through mutations, loss of heterozygosity, deletions, or epigenetic modifications have been the major factors in its development, local invasion and local metastasis (1,4).

The homeobox genes encode transcription factors that acts either by activating or repressing downstream target genes essential to cell growth and differentiation. It is estimated that the human genome includes at least 200 homeobox genes, 39 of which belong to the *HOX* family. These genes are functionally important during embryonic morphogenesis and also regulate the adult tissue architecture, identity and homeostasis, cell-cell interactions and cell-extracellular matrix interactions (5).

In cancer, normal *HOX* gene expression is disrupted, affecting various pathways that promote tumorigenesis and metastasis, including the activation of anti-apoptotic pathways and suppression of differentiation (6). *HOX* genes have been found to be aberrantly expressed in a variety of solid tumors such as lymphoma (7,8), melanoma (9), breast (10,11), endometrial (10), liver (12), lung (13,14), thyroid (15) and esophagus cancer (16). Aberrant expression of *HOX* genes was also observed in OSCC; however, how they contribute to oral cancer phenotype and its tissue-specific features remains unclear (17–19).

Detection of OSCC is currently based on expert clinical examination and histological analysis of suspicious areas, but it may be undetectable in hidden sites. Therefore, sensitive and specific biomarkers for OSCC may be helpful to screening high-risk patients (20). While several studies proposed the identification of gene expression patterns in head and neck cancer (21–24), just a few investigated the differential expression profile of homeobox genes family in OSCC (17,25–27) as well as their correlation to tumor behavior, clinical parameters and survival rates (25,26), obtaining significant results.

Thus, the purpose of the present study was to search for distinct pattern of homeobox gene expression through a genome-wide analysis. Some up- and down-regulated homeobox genes were chosen for further validation by qRT-PCR and correlated with prognosis. Up-regulated homeobox genes were also validated on OSCC cell lines.

## Materials and methods

### Tissue samples

Specimens were obtained during surgical resection from patients aged ≥40 years, admitted for diagnosis and treatment at Arnaldo Vieira de Carvalho Cancer Institute, Hospital Heliópolis and Hospital das Clínicas of São Paulo University Medical School. Histopathological diagnosis was performed according to the WHO classification of tumors by the Department of Pathology of each Institution. Clinicopathological staging was determined by the TNM classification of the IUCC (28). The study was approved by the Ethics Committee of each Institution and was based on the criteria of the Helsinki convention.

Fresh surgical samples of primary OSCC and their corresponding non-neoplastic margin tissues were immediately snap-frozen in liquid nitrogen upon surgical removal. After histological confirmation, all tissue samples were checked prior to RNA extraction so that each OSCC sample contained at least 70% tumor cells and the corresponding surgical margins were reported as ‘tumor-free’. GENCAPO (Head and Neck Genome Project) Consortium was responsible for sample collection and initial processing, clinical data collection, providing of histopathological analysis of tissue samples, and informed consent acquisition of each patient.

### Cell lines and cell culture

SCC-4, -9, -15 and -25 (OSCC cell lines) were obtained from the American Type Culture Collection (ATCC, Manassas, VA, USA) and kindly provided by Professor Ricardo Della Coletta (School of Dentistry, UNICAMP). OSCC as well as HaCat cell lines were grown as described previously (25). Normal oral keratinocytes (NOK) were obtained from oral epithelial fragments under enzymatic digestion method, kindly provided by Dr Maria Fatima Guarizo Klingbeil (29).

### RNA extraction and cDNA synthesis

Total cellular RNA was extracted using TRIzol^®^ Reagent (Invitrogen, Carlsbad, CA, USA) according to the manufacturer's instructions and the RNA integrity was evaluated based on the intensity of 28S and 18S rRNA bands in 1% agarose gels and on A260/280 ratio between 1.8 and 2.0.

RNA obtained from tissue samples (1 μg) and cell lines (4 μg) was reverse transcribed to single-stranded cDNA using High Capacity cDNA Reverse Transcription kit (Applied Biosystems™, Foster City, CA, USA) and Superscript III™ with oligo(dT) primers and RNase OUT (Invitrogen), respectively, after incubation with DNAse I (Invitrogen).

### Microarray hybridization

Ten tissue samples of primary OSCC of tongue and floor of the mouth, as well as a pool of non-neoplastic surgical margins were used for microarray analysis. Experiments were carried out as described in Severino *et al* (30) using CodeLink Whole Genome Bioarrays (GE Healthcare, Piscataway, NJ, USA) representing 55,000 human transcripts and arrays were scanned on a GenePix 4000B Array Scanner (Axon Instruments), according to the recommended scanning procedures and settings. The data were treated with Code-Link feature extraction software v.4.0. A normalized signal for each transcript was obtained through quantile normalization (31). For global homeobox gene expression visualization, a hierarchical clustering using the Euclidean distance and the average linkage algorithm was performed (MeV^®^ MultiExperiment Viewer software version 4.1, Boston, MA, USA) (32,33).

Individual homeobox gene expression profile in OSCC samples and their respective non-neoplastic oral tissues were compared with each other. Differentially expressed genes were identified by calculating the ratio of the mean normalized fluorescence values obtained from each sample group. Results were expressed as fold variation, and genes displaying greater than 2-fold changes in transcript abundance in all tumor samples were selected. The array design and raw data files are available at the Gene Expression Omnibus database (GEO) under the accession number GSE9792. The most up-regulated homeobox genes were selected and analyzed by qRT-PCR.

### qRT-PCR

Samples of OSCC tissues and non-neoplastic margins were assessed for the expression levels of selected homeobox genes (*HOXA5*, n=36; *HOXD10* and *HOXD11*, n=39). The same was performed for all cell lines described above. Endogenous housekeeping gene coding for the hypoxanthine guanine phosphoribosyltransferase gene (*HPRT*; NM_000194.2; F: ccaccaccctgttgctgta and R: tcccctgttgactggtcat; 119 bp) was used for data normalization and relative quantification was performed using relative standard curve analysis with a 7500 real-time PCR System (Applied Biosystems, Foster City, CA). Amplification of specific PCR products was detected using the SYBR Green PCR Master Mix (Applied Biosystems) according to the manufacturer's protocol. Each run was completed with a melting curve analysis to confirm the specificity of amplification and lack of primer dimers. *HOXA5* primer sequences (NM_019102.2) were designed from a specified exon-exon junction of the gene of interest (F: gcgcccgccatgtcctac and R: agaccggcgcctgggcc; 151 bp), using GeneTool 2.0 software (Biotools, Edmonton, AB, Canada). *HOXD10* (NM_002148.3) and *HOXD11* (NM_021192.2) primers were purchased from SuperArray Biosciences™ (Frederick, MD, USA, RT2 qPCR Primer Assay, cat# PPH11616A; 147 bp and PPH19882A; 155 bp, respectively). All qPCR reactions were performed in a total volume of 25 μL, containing 1 μL of cDNA sample, 10 ρmol of each primer (400 nM) or 1.0 μL of RT2 PCR primer set and 12.5 μL of SYBR Green Master Mix^®^ (Applied Biosystems). The thermal cycling was carried out by starting with 95˚C for 10 min hold, followed by 40 amplification cycles of 95˚C for 10 sec and 60˚C for 1 min.

### Statistical analysis

The differences in gene expression levels in tissue samples and OSCC cell lines were analyzed by Wilcoxon non-parametric test and one-way ANOVA with Tukey's post-test, respectively. The differential expression of *HOXA5*, *HOXD10* and *HOXD11* in tissue samples was divided into two groups (higher versus lower) according to the value obtained from qRT-PCR. The cut-off value was set up at the median expression level. Fisher's exact test was used to estimate statistical difference between *HOX* genes expression levels and clinicopathological parameters such as mean age, tumor location, tumor size-pT, nodal metastasis-pN, pathological grade, lymphatic and/or perineural invasion and recurrence. For this analysis, only OSCC samples paired with their respective non-neoplastic margins in which *HOX* genes exhibited detectable expression by qRT-PCR were used (*HOXA5*, n=35; *HOXD10* and *HOXD11*, n=34). Kaplan-Meier product-limit estimation with log-rank (P<0.05) was used for survival analysis from life-time data according to gene expression levels, in view of investigating the most relevant gene or gene sets to predict tumor prognosis, as well as anatomic site, histopathological grade of differentiation, perineural and/or lymphatic infiltration. Overall survival was defined as time from surgery to the day of death or last follow-up. Statistical package GraphPad Prism 5 (GraphPad Software, Inc., CA, USA) was used for the statistics.

## Results

### HOX genes expression patterns in OSCC tissues and cell lines

**Figure 1:**
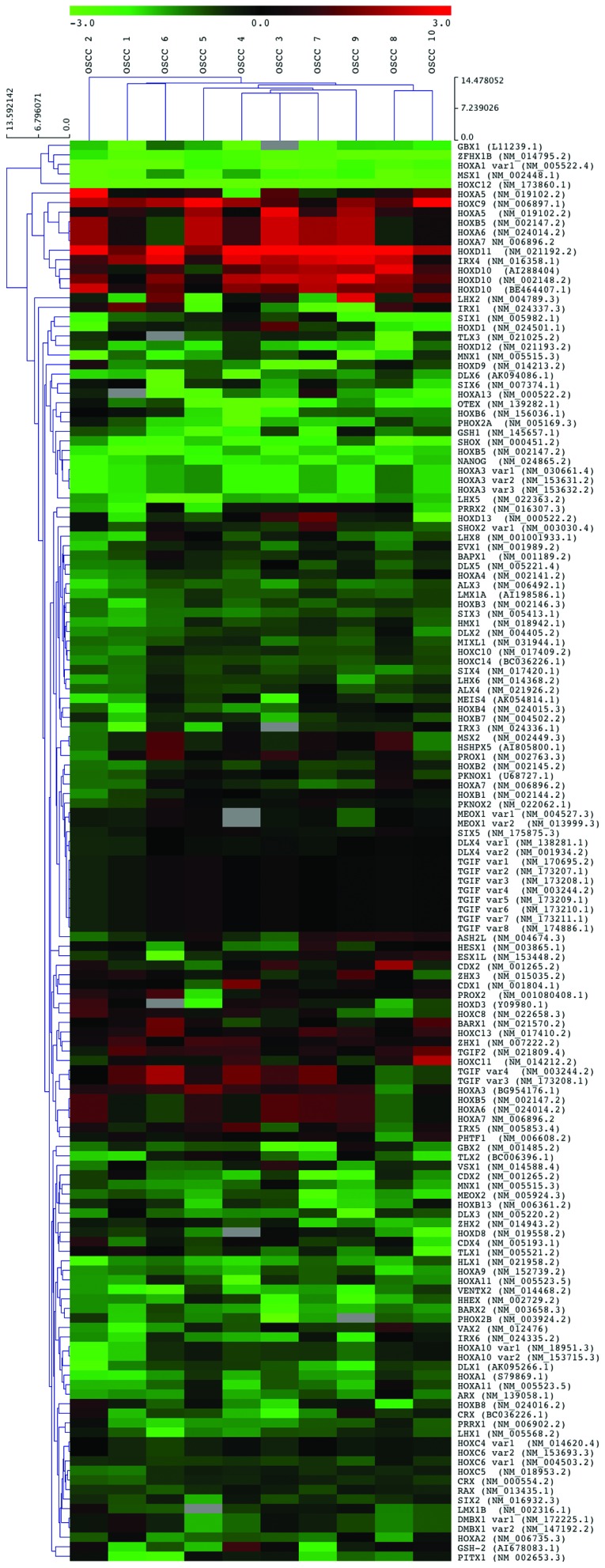
Hierarchical cluster diagram of differential homeobox gene expression in OSCC samples. Gene expression levels in non-neoplastic margins were used as baseline. Data are visualized colorimetrically with heat plots, ‘red’ representing elevated gene expression and ‘green’ decreased gene expression.

The profile of expression of the homeobox genes through microarray analysis was performed and Fig. 1 shows up- and down-regulated homeobox genes in tumors in relation to their non-tumoral counterparts. A general analysis of microarray data revealed that, among 147 homeobox genes evaluated, two sets of homeobox genes with relatively homogeneous expression patterns were found, in which a set of homeobox genes were predominantly down-regulated while the other set was predominantly up-regulated. The other homeobox genes showed differential expression patterns greatly variable between OSCC tissue samples.

The set of at least 2-fold up-regulated homeobox genes in OSCC samples included 6 genes (Table I) while the set of at least 2-fold down-regulated homeobox genes included 34 genes. Considering the technique resolution (noise) as well as the most viable clinical application, the set of homeobox genes predominantly up-regulated was chosen for validation.

**Figure 2:**
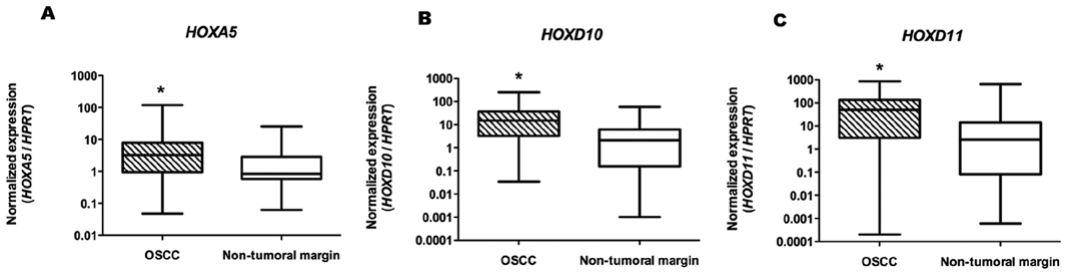
Normalized expression of *HOXA5*, *HOXD10* and *HOXD11* transcripts by qRT-PCR in OSCC samples and non-tumoral margins. The full line corresponds to the median value for each group. Asterisk indicates statistically significant difference between OSCC and non-tumoral samples (p<0.05, Wilcoxon)..

Among the 6 up-regulated homeobox genes, *HOXA5*, *HOXD10* and *HOXD11* showed the highest expression levels (average: 8.65-fold). These genes were selected for further analysis and validation by qRT-PCR in a larger cohort of patients (*HOXA5*, n=36; *HOXD10* and *HOXD11*, n=39) and OSCC cell lines. When analyzing the frequency of detectable gene expression per tumor sample (presence/absence), no detectable expression of *HOXA5* was observed in one case, while absence of amplification of either *HOXD10* or *HOXD11* transcripts was observed in two cases. However, when comparing OSCC tissue samples with the paired non-neoplasic margins (*HOXA5*, n=35; *HOXD10* and *HOXD11*, n=34), high mRNA expression levels of *HOXA5*, *HOXD10* and *HOXD11* were consistently detected by qRT-PCR, with statistical significance (P<0.001, P<0.001 and P<0.005, respectively), as shown in Table II and Fig. 2.

Sample characterization and the correlation of *HOXA5*, *HOXD10* and *HOXD11* expression levels with clinicopathological features and disease outcome were examined and are shown on Table III. In general, there was no significant association between *HOXA5*, *HOXD10* and *HOXD11* expression levels and age group, tumor location, pTNM classification, pathological grade, lymphatic and/or perineural invasion and local recurrence. However, although not statistically significant, moderately differentiated tumors showed higher levels of *HOXD11* expression (P=0.08).

**Figure 3:**
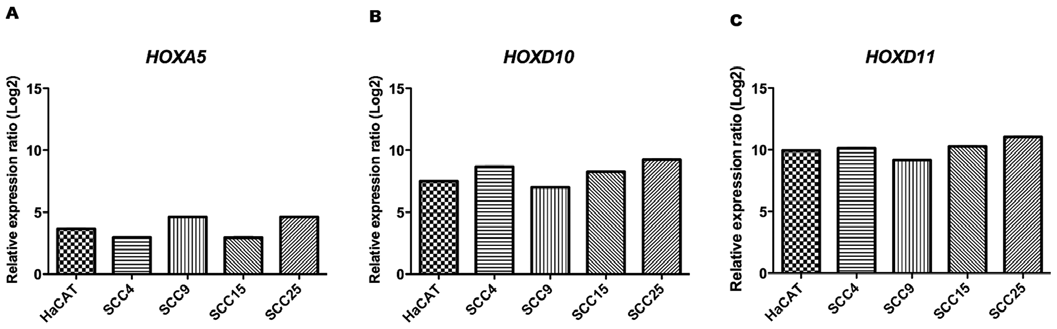
Relative expression ratio (log_2_) of *HOXA5*, *HOXD10* and *HOXD11* mRNA expression analysis by qRT-PCR in HaCAT and OSCC cell lines (SCC4, 9, 15, 25). Significant up-regulation of these genes was observed in all cell lines compared with that in NOK (p<0.001, one-way ANOVA).

Additionally, the status of *HOXA5*, *HOXD10* and *HOXD11* mRNA expression levels were evaluated in HaCat and OSCC cell lines by qRT-PCR. Relative quantitation analysis revealed that these genes were significantly up-regulated in all cell lines when compared to NOK (calibrator sample, gene expression level =1), showing mean levels of 4-fold, 8-fold and 10-fold higher (P<0.001) than NOK regarding *HOXA5*, *HOXD10* and *HOXD11* expression, respectively (Fig. 3). These data confirm that the up-regulation of the genes observed in OSCC cell lines were also in accordance to the microarray analysis of tissue OSCC samples.

### HOXA5 expression level is associated with the survival rate

**Figure 4:**
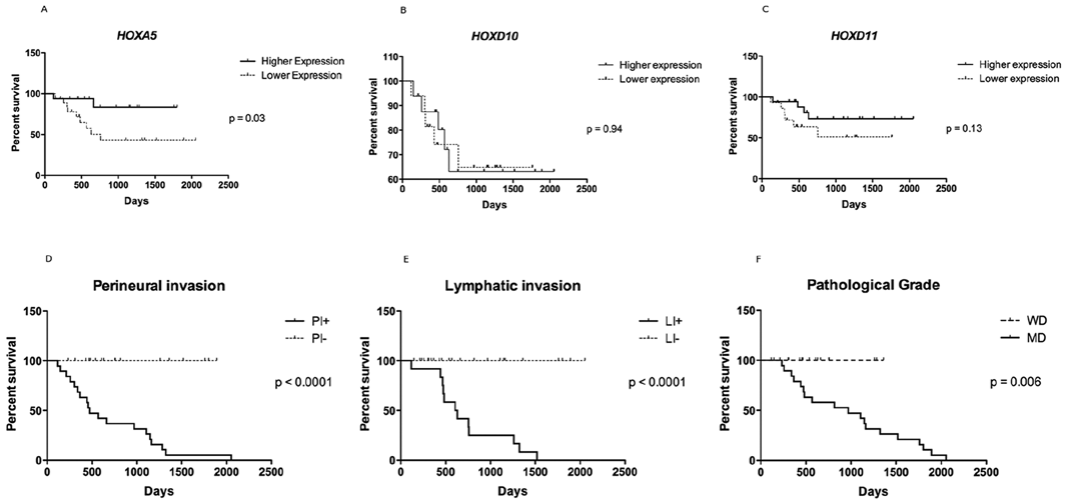
Survival proportions (log-rank test) of OSCC patients according to *HOXA5* (A), *HOXD10* (B) and *HOXD11* (C) expression, as well as to perineural invasion (D), lymphatic invasion (E) and pathological grade (F). (LI, lymphatic invasion; PI, perineural invasion; WD, well differentiated; MD, moderately differentiated).

Although *HOXA5*, *HOXD10* and *HOXD11* genes were all up-regulated, a cut-off value for the expression level was set up at the median expression level, defining samples with higher and lower expression levels. The P-value for the survival curve, determined by the log-rank test, showed statistically significant difference in the survival rates only between higher and lower expressions of *HOXA5* (P=0.03). Patients with higher expression of *HOXA5* (the 5-year survival rate of 16 patients was 83.3%) had much more favorable prognosis than those with lower expression (the 5-year survival rate of 18 patients was 43%). *HOXD10* and *HOXD11* expression was not related to overall survival (Fig. 4A–C).

A higher overall survival rate was observed in cases presenting no lymphatic as well as perineural infiltration (P<0.0001) and those microscopically classified as well differentiated (P=0.006) tumors (Fig. 4D–F).

## Discussion

Since many homeobox genes normally expressed by embryonic tissues are aberrantly activated or re-expressed in tumors, some speculate that these genes may act as oncogenes in solid tumors. However, homeobox genes may also be down-regulated in malignant cells of tissues in which a particular gene is normally expressed in a complete differentiated state, what is consistent with a tumor suppressor gene.

In the present study, the difference in homeobox gene expression levels were investigated in OSCC tissue samples in relation to their non-tumoral counterparts as well as in OSCC and NOK cell lines. Regarding control tissue samples, field cancerization (34) is a widely accepted theory meaning that the margin mucosa may present some tumor-related molecular changes despite its normal morphological appearance. However, non-tumoral margins were undertaken as control samples in the present study in order to avoid questioning if that the observed differences in gene expression could be related to possible individual variability. Inter-individual differences in phenotype, whether associated with disease or not, are generally assumed to reflect inter-individual differences in the expression of genes. According to Turan *et al* (35) one of the most surprising observations to emerge from human transcriptome profiling is the very high level of inter-individual variability found in steady state mRNA levels of many genes. Moreover, the ideal control tissue in a study should be obtained from the same patient and from the same tumor site (in our case tongue and floor of the mouth).

In view of the above, consistent differences in expression levels of *HOXD11*, *HOXD10*, *HOXA5*, *IRX4*, *HOXC9* and *HOXA6* were observed in OSCC samples in relation to the non-tumoral counterparts after microarray analysis. *HOXA5*, *HOXD10* and *HOXD11* showed the highest expression levels and their up-regulation was then validated by qRT-PCR in tumor samples as well as in cell lines. Levels of *HOXA5* below the cut-off value (lower expression) were also associated with poor prognosis of OSCC.

Others also identified transcripts studied herein as differentially expressed in primary tumors from sites other than oral cavity. Evidence of altered expression of *HOXD10* is strong in breast and endometrial cancer, in which *HOXD10* expression is progressively reduced in epithelial cells as malignancy increases. Also, after restoring *HOXD10* expression in malignant breast tumor cells, cell migration was significantly impaired and their ability to form tumors in mouse xenografts was inhibited (10). Reddy *et al* (36) observed that loss of *HOXD10* expression is related to micro-RNA miR-7 and contributes to increased invasiveness in breast cancer. While these findings suggest that *HOXD10* has tumor-suppressive functions for mammary epithelial cells, a different scenario is observed for esophageal (37) and oral cancer.

In the present study, although the expression levels of *HOXD10* did not influence the overall survival rate, it was significantly up-regulated in OSCC samples (median value >8-fold) in relation to non-tumoral tissues as well as in OSCC cell lines. This is in agreement with Hassan *et al* (17) who revealed significantly higher expression levels of *HOXD10* in OSCC compared to those in normal oral mucosa, as well as higher expression levels in dysplasia tissues compared to normal oral mucosa tissues, suggesting that *HOXD10* expression sequentially alters from normal mucosa, to dysplasia and OSCC.

A similar heterogeneous pattern is observed regarding *HOXD11* expression. This gene seems to be silenced in breast cancer (38), ovarian cancer (39) and melanoma (9), suggesting that a specific methylation pattern of a group of genes, involving *HOXD11*, may be useful as diagnostic and prognostic biomarkers. On the other hand, *HOXD11* is transcribed in gastric carcinoma in an abnormal manner suggesting an important role in the development of this disease (40). The same occurs in OSCC, as observed in the present study and others (17). Here, a significantly higher expression level of *HOXD11* was detected by qRT-PCR in OSCC tissue samples (median value of 5-fold) and OSCC cell lines, although with no correlation to survival rates.

*HOXA5* also presents the same variable pattern of expression. In primary breast carcinoma, *HOXA5* has also been implicated as a tumor suppressor gene since its expression is lost in >60% breast cancer cell lines and primary tumors (41,42). In agreement, *HOXA5* is also down-regulated in the vast majority of non-small cell lung cancer, which is associated with a borderline significantly worse survival in patients with stage I disease (43). Nevertheless, Yang *et al* (44) observed that homeobox genes from cluster A (*HOXA4*, *HOXA5*, *HOXA7*, *HOXA9* and *HOXA13*) were highly expressed in gastric cancer cell lines and suggested that the mechanism of gastric carcinogenesis possibly involves specific chromosomal rearrangement and up-regulation of *HOX* genes. Similar findings were observed here, showing the validation by qRT-PCR of *HOXA5* higher expression in OSCC tissues (median value >3-fold) and cell lines.

There are few studies that investigated the differential expression profile of homeobox genes in OSCC (17,25–27) and their correlation with tumor behavior, clinical parameters and survival rates (25,26). De Souza Setúbal Destro *et al* (25) showed the overexpression of *HOXB7* in tumor samples and its association with tumor size, lymph node state and clinical stage of disease, reflecting a lower overall and disease-free survival rates. Yamatoji *et al* (26) also associated *HOXA10* overexpression with tumor differentiation grade, aggressiveness and prognosis, describing *HOXA10* up-regulation as a putative prognostic marker of lower overall and disease-free survival rates.

As expected, a higher overall survival rate was observed in the present study for cases presenting no lymphatic as well as perineural infiltration and those microscopically classified as well differentiated tumors. Considering that *HOXA5*, *D10* and *D11* were significantly over-expressed in OSCC samples, we could expect to correlate these gene expression levels with some of those clinicopathological features of the tumors. A possible explanation for the lack of correlation may be due to the fact that, except from perineural invasion, histopathological grade and lymphatic invasion are considered limited independent prognostic factors (45). Although WHO (46) recommends the use of the categories well-, moderately- and poorly-differentiated this grading system usually depends on a subjective assessment, being considered by most authorities as a poor indicator of outcome and response to treatment (47–50). Also, the prognostic value of lymphovascular invasion is questionable since it is difficult to define and recognize with certainty in routinely stained tissues (50).

The results presented here and by others (17) support the hypothesis that aberrant expression of *HOX* genes is associated with the development of OSCC. Nevertheless, although the expression levels of *HOXD10* and *HOXD11* did not influence overall survival rates in the present study, a significant association was found for *HOXA5*. Our results revealed that patients with higher expression of *HOXA5* had much more favorable prognosis than those with lower expression.

It was demonstrated that reduction or loss of *HOXA5* expression correlates with reduced p53 levels in breast tumors, suggesting that loss of *HOXA5* expression is an important step in tumorigenesis (41). In addition, there is coordinated loss of both HOXA5 and retinoic acid receptor (RARb) expression during neoplastic transformation and progression in a breast epithelial cell model. Knockdown of *HOXA5* expression partially abrogates retinoid-induced apoptosis and promotes cell survival upon retinoic acid treatment. These results strongly suggest that HOXA5 acts directly downstream of RARb and may contribute to retinoid-induced anticancer and chemopreventive effects (51).

Target genes for the homeobox transcription factors are either homeobox genes themselves or other genes that are critical to controlling cell division, adhesion and migration, morphological differentiation and apoptosis. Currently, there are no well-established specific target genes for the studied homeobox genes. From what is known so far, homeobox proteins interact with numerous regulatory pathways, including FGF (fibroblastic growth factor), BMP (bone morphogenetic protein), retinoic acid, sex steroid signaling (52) and proteins involved in cell-matrix interaction, such as integrins and ICAM (intercellular adhesion molecule) (53). In gastric cancer, *HOXD11* is expected to exert a regulating role in αV integrin gene, even if its expression pattern in tumors contrasts with the functions that this protein seems to have in neoplastic cells, mainly promoting cell migration and survival (40).

In conclusion, this study is the first to investigate the expression profile of homeobox genes in OSCC based on differentially expressed genes identified through a microarray genome-wide screening. The present results confirmed the presence of three significantly up-regulated (>4-fold) homeobox genes (*HOXA5*, *HOXD10* and *HOXD11*) in OSCC that may play a significant role in the pathogenesis of these tumors and that deserve further functional investigation to understand the cellular processes involved. Moreover, it was shown that lower levels of *HOXA5* predict poor prognosis for patients with OSCC after surgery, suggesting that this gene may be a novel candidate for development of OSCC therapeutic strategies.

## Figures and Tables

**Table I:** Up-regulated genes (≥2-fold) in OSCC samples relative to non-neoplastic tissue as indicated by microarray analysis.

Gene	Mean fold-change	NCBI access
*HOXD11*	8.88	NM_021192.2
*HOXD10*	8.88	NM_002148.3
*HOXA5*	8.21	NM_019102.2
*IRX4*	4.15	NM_016358.2
*HOXC9*	3.82	NM_006897.1
*HOXA6*	2.69	NM_024014.2

**Table II:** Median (range) expression levels of *HOXA5*, *HOXD10* and *HOXD11* homeobox genes by qRT-PCR in OSCC samples and non-tumoral margins.

Gene	OSCC (min-max)	Non-tumoral margin (min-max)	P-value^a^
*HOXA5*	3.24 (0.05–119.70)	0.85 (0.06–25.76)	P<0.001
*HOXD10*	15.09 (0.03–251.00)	2.13 (0.001–58.25)	P<0.001
*HOXD11*	50.29 (0.0002–855.60)	2.51 (0.0006–639.00)	P<0.005

a P-value according to Wilcoxon non-parametric test.

**Table III:** Association of *HOXA5*, *HOXD10* and *HOXD11* homeobox genes expression levels with OSCC clinicopathological features (P-value according to Fisher's exact test).

Clinicopathological features	No. of cases^b^	HOXA5 expression			HOXD10 expression			HOXD11 expression		
		Higher	Lower	P-value	Higher	Lower	P-value	Higher	Lower	P-value
Age										
40–60 years	25	11	15	0.26	14	11	0.70	13	12	1.00
>60 years	9	6	3		4	5		5	4	
Tumor location										
Tongue	12	6	7	1.00	5	7	0.47	6	6	0.72
Floor of mouth	22	11	11		13	9		13	9	
pT classification										
T1	3	3	1	1.00	2	1	0.73	2	1	1.00
T2	11	4	7		6	5		4	7	
T3	13	6	7		7	6		11	2	
T4	7	4	3		3	4		2	5	
pN classification										
N+	14	7	8	1.00	8	6	0.73	6	8	1.00
N0	20	10	10		10	10		13	7	
Pathological grade										
Well differentiated	16	9	8	0.73	8	8	1.00	6	10	0.08
Moderately differentiated	18	8	10		10	8		13	5	
Lymphatic invasion (LI)^a^										
LI−	21	11	11	0.72	12	9	0.73	12	9	1.00
LI+	12	5	7		6	6		7		
Perineural invasion (PI)										
PI−	16	7	9	0.73	10	6	0.32	9	7	1.00
PI+	18	10	9		8	10		10	8	
Local recurrence										
Present	6	4	3	0.68	2	4	0.38	2	4	0.36
Absent	28	13	16		16	12		17	11	

a Missing data of one patient.

b The number of paired cases with detectable expression by qRT-PCR was 35 OSCC samples for *HOXA5* and 34 OSCC samples for *HOXD10* and *HOXD11* transcripts.

## Appendix

The GENCAPO (Head and Neck Genome) Project members are the following: Cury PM^3^, De Carvalho MB^7^, Dias-Neto E^1^, Figueiredo DLA^8^, Fukuyama EE^5^, Góis-Filho JF^5^, Leopoldino AM^12^, Mamede RCM^8^, Michaluart-Junior P^1^, Moreira-Filho CA^1^, Moyses RA^1^, Nóbrega FG^4^, Nóbrega MP^4^, Nunes FD^6^, Ojopi EPB^1^, Okamoto OK^11^, Serafini LN^8^, Severino P^2^, Silva AMA^7^, Silva WA Jr^8^, Silveira NJF^10^, Souza SCOM^6^, Tajara EH^3^, Wünsch-Filho V^9^, Zago MA^8^, Amar A^7^, Arap SS^1^, Araújo NSS^1^, Araújo-Filho V^1^, Barbieri RB^7^, Bandeira CM^4^, Bastos AU^7^, Bogossian AP^4^, Braconi MA^4^, Brandão LG^1^, Brandão RM^8^, Canto AL^4^, Carvalho-Neto PB^7^, Casemiro AF^7^, Cerione M^5^, Cernea CR^1^, Cicco R^5^, Chagas MJ^4^, Chedid H^7^, Chiappini PBO^7^, Correia LA^7^, Costa A^9^, Costa ACW^7^, Cunha BR^3^, Curioni OA^7^, Dias THG^1^, Durazzo M^1^, Ferraz AR^1^, Figueiredo RO^9^, Fortes CS^9^, Franzi SA^7^, Frizzera APZ^3^, Gallo J^1^, Gazito D^7^, Guimarães PEM^1^, Gutierres AP^7^, Inamine R^9^, Kaneto CM^8^, Lehn CN^7^, López RVM^9^, Macarenco R^4^, Magalhães RP^1^, Martins AE^7^, Meneses C^4^, Mercante AMC^7^, Montenegro FLM^1^, Pinheiro DG^8^, Polachini GM^3^, Porsani AF^7^, Rapoport A^7^, Rodini CO^6^, Rodrigues AN^9^, Rodrigues-Lisoni FC^3^, Rodrigues RV^3^, Rossi L^7^, Santos ARD^8^, Santos M^7^, Settani F^5^, Silva FAM^12^, Silva IT^8^, Silva-Filho GB^1^, Smith RB^1^, Souza TB^7^, Stabenow E^1^, Takamori JT^7^, Tavares MR^1^, Turcano R^1^, Valentim PJ^5^, Volpi EM^1^, Xavier FCA^6^, Yamagushi F^5^, Cominato ML^5^, Correa PMS^4^, Mendes GS^5^, Paiva R^5^, Ramos O^1^, Silva C^1^, Silva MJ^5^ and Tarlá MVC^8^.

Affiliations: ^1^School of Medicine, USP, São Paulo; ^2^The Albert Einstein Jewish Teaching and Research Institute (IIEP), São Paulo; ^3^School of Medicine of São José do Rio Preto; ^4^São José dos Campos Dental School, UNESP; ^5^Cancer Institute Arnaldo Vieira de Carvalho, São Paulo; ^6^School of Dentistry, USP, São Paulo; ^7^Heliópolis Hospital, São Paulo; ^8^School of Medicine of Ribeirão Preto, USP; ^9^School of Public Health, USP, São Paulo; ^10^University of Vale do Paraíba, São José dos Campos; ^11^School of Medicine, UNIFESP, São Paulo; ^12^Faculty of Pharmaceutical Sciences of Ribeirão Preto, USP, SP, Brazil.
